# Latent profile analysis of psychological needs thwarting in Chinese school teachers: longitudinal associations with problematic smartphone use, psychological distress, and perceived administrative support

**DOI:** 10.3389/fpubh.2023.1299929

**Published:** 2023-12-18

**Authors:** Xiao-Ling Liao, Cui-Hong Cao, Jeffrey H. Gamble, Ling-Ling Li, Xing-Yong Jiang, Cun-Xu Bo, I-Hua Chen

**Affiliations:** ^1^International College, Krirk University, Bangkok, Thailand; ^2^School of Foreign Languages, Shandong Women’s University, Jinan, China; ^3^Faculty of Education, Qufu Normal University, Qufu, China; ^4^Department of English, National Changhua University, Changhua, Taiwan; ^5^No.1 Senior High School, Xinjian District of Nanchang City, Nanchang, China; ^6^Yangan Primary School of Qionglai City, Qionglai, China; ^7^Shandong Provincial Institute of Education Sciences, Jinan, China; ^8^Chinese Academy of Education Big Data, Qufu Normal University, Qufu, China

**Keywords:** psychological needs thwarting, problematic smartphone use, psychological distress, perceived administrative support, latent profile analysis

## Abstract

**Introduction:**

In light of the significant impact that teachers have on education quality and student growth, their mental health warrants special attention. With the increasing popularity of Information and Communication Technology (ICT) and the rise of online teaching during the pandemic, teachers have become a group prone to developing problematic smartphone use (PSU). Psychological need thwarting (PNT) has been shown to be closely related to PSU, psychological distress, and perceived administrative support. However, most previous studies have adopted a variable-centered approach, which may overlook the possibility that the three basic needs are not closely associated and could form distinct profiles. Therefore, this study aims to apply latent profile analysis to identify different PNT profiles and their associations with PSU, psychological distress, and perceived administrative support.

**Methods:**

A longitudinal survey was conducted using convenience and purposive sampling methods. The survey involved 1,642 primary and middle school teachers working in China over a two-month interval, with the first assessment in November 2021 (Time 1) and the second in January 2022 (Time 2).

**Results:**

The results indicate that a three-profile model, intricately based on the PNT data gathered at Time 1, is most optimal: Class 1 is labeled as ‘High autonomy-High competence and Moderate relatedness thwarting’, Class 2 as ‘High autonomy-High competence and High relatedness thwarting’, and Class 3 as ‘Low psychological needs thwarting’. Distinct associations were observed among the three profiles concerning PSU, psychological distress, and perceived administrative support. Specifically, in terms of PSU, the score of Class 2 was higher than Class 1, with that of Class 3 being the lowest at Time 1, while at Time 2 no significant difference was found between any two of these three groups; in terms of distress, the scores of the three profiles were arranged from high to low as Class 2, 1, and 3 at both time points; and in terms of perceived administrative support, the order was just the opposite, with 3, 1, and 2 from high to low at both Time 1 and Time 2.

**Conclusion:**

Notably, the consistent ranking of the three classes in terms of psychological distress and administrative support suggests a lasting influence of PNT. Future studies should explore this enduring impact further by employing additional longitudinal data sets and examining potential mediators or moderators beyond the current study’s scope.

## 1 Introduction

The psychological well-being of teachers holds significant importance in the educational system. Compromised health can impact not only their ability to deliver quality instruction and address students’ needs but also increase the workload for other staff (1). In this context, studies like those by Chen et al. (2) have highlighted how teachers’ negative moods, such as fear, can influence their students. A related and growing concern is the rise of Problematic Smartphone Use (PSU) among teachers, particularly due to the increased reliance on Information and Communication Technology (ICT) in education (3, 4). This issue has become more pronounced during the pandemic, where the abrupt shift to online teaching has necessitated that teachers spend extensive time on smartphones, leading to PSU (5–7). This excessive smartphone use, while a direct consequence of the pandemic-induced changes, also feeds into a cycle of psychological distress among teachers (8, 9).

To address these interconnected issues, this study employs the concept of Psychological Need Thwarting (PNT) (10), recognized both as a marker of psychological distress (11, 12) and as a risk factor for PSU (13, 14). The study uses PNT as a framework to explore the intricate relationship between PSU, psychological distress, under the topic of mental health of teachers. Crucially, we also delve into the role of administrative support during the pandemic’s transition to online teaching. This period highlighted the importance of administrative support, not only as a critical resource for teachers (15) but also as a potential source of PNT (16). Inadequate support can lead to increased PNT (17), exacerbating the challenges of PSU (18) and psychological distress (19). In the following sections of the manuscript, we commence with a detailed discussion of PNT. This establishes a foundational understanding, which is essential for later examining its connection with PSU and psychological distress among teachers. Simultaneously, the study explores how varying levels of administrative support during the emergency transition to online teaching either exacerbated or alleviated these challenges, a topic intrinsically linked to PNT. By including PSU, psychological distress, and administrative support in our analysis, we aim to provide a more comprehensive understanding of the factors affecting teachers’ mental health in the digital age, particularly under the unique strains introduced by the pandemic.

### 1.1 Psychological need thwarting and related studies

Rooted in Self-Determination Theory (SDT), PNT is characterized by the perception that basic psychological needs are actively undermined by various obstacles and damages (10). According to SDT, needs define innate psychological nutrients necessary to maintain psychological integrity, growth, and well-being, in which three basic psychological needs are identified: autonomy, competence, and relatedness (20–22). Autonomy reflects a willingness to be responsible for one’s experiences and behaviors, aiming for an integrated and authentic self. Competence pertains to a sense of effectiveness and the ability to interact with one’s environment and fulfill responsibilities. Relatedness involves feeling connected to others and being valued by them (22). Also, this theory holds that engaging in interesting activities, exercising capacities, and establishing connections with others are essential parts of the human adaptive design (22). Satisfying these psychological needs is crucial for motivation and well-being (23, 24).

Conversely, thwarting these needs leads to experiences of PNT (11) and has a more negative consequences on affect than psychological need satisfaction (20, 25, 26). Yet, PNT has been less explored than psychological need satisfaction (27). Studies have linked PNT to student disengagement (28), depression in weight management contexts (12), and negative outcomes in sports (10). Gunnell et al. (29) also found that PNT predicted ill-being in physical activity contexts.

However, research of PNT focusing on teachers in social professions, is limited. Most studies have centered on physical education teachers (1, 30). Bartholomew et al. (1) found that each need thwarting was positively associated with burnout and job pressure. Another study linked burnout with teachers’ perceptions of need thwarting (30). Some research has also explored teachers’ PNT in online teaching contexts (11, 20). However, most studies have used variable-centered models, like structural equation modeling, with few adopting a person-centered approach.

Empirical evidence suggests that the three basic needs are not highly correlated (17). For instance, the correlation coefficients between relatedness thwarting and autonomy thwarting, and between relatedness thwarting and competence thwarting, are below 0.5 (17). This indicates potential for distinct profiles. Given this, our primary aim is to determine if distinct teacher groups emerge based on PNT using Latent Profile Analysis (LPA). If yes, how many groups will be divided into?

### 1.2 The association of psychological need thwarting with PSU, psychological distress, and administrative support among teachers

As an emerging construct in teacher health research, PNT offers insights into how the thwarting of these three basic needs impacts an individual’s mental health and behaviors (8). PNT has been linked to several factors, including PSU (31), psychological distress (10, 12), and administrative support (11). PSU has been identified as one factor associated with PNT. With their convenience and portability, smartphones have become integral to daily life, facilitating interpersonal communication, socialization, and knowledge acquisition. However, excessive and uncontrolled smartphone use, termed PSU, can have detrimental effects on users’ physical and mental health (32, 33). Existing research has discussed the impact of PSU on teachers and students. Adopting a mixed method, Varanasi et al. (4) found that though smartphones were helpful for teaching, problematic use of them also significantly predicted teachers’ burnout. Butt and Arshad studied the basic psychological needs of university students with PSU and found that those with PSU experienced higher levels of need thwarting, often using smartphones to fulfill their relatedness needs in social environments (31). In addition, PSU was proved to exert an indirect positive impact on teachers’ psychological distress with PNT being a mediator in online teaching contexts (8). Unmet psychological needs have been identified as significant predisposing factors for adolescents’ PSU (33, 34). Besides, the phenomenon that people with negative feelings such as PNT, depression are easy to subject to PSU can be explained by the Compensatory Internet Use Theory (CIUT), which denotes that when faced with negative life circumstances, people may resort to excessive smartphone use to relieve their negative feelings (35).

In addition to PSU, previous studies have also highlighted the negative effects of PNT on psychological distress, such as depression (10, 12), stress, and anxiety (1). Vansteenkiste et al. (21) noted a direct association between PNT and students’ symptoms of ill-being and distress. Similarly, Nishimura and Suzuki (36) found that the thwarting of each need predicted symptoms of ill-being, specifically, depressed affect. Gilbert et al. (37) also reported a strong predictive relationship between need thwarting and students’ psychological distress. Besides focusing on students, Chen et al. (20) evaluated the persistent and long-term impact of PNT of online teaching on teachers’ distress.

Another focal factor of this study is perceived administrative support. The term was defined by Borman and Dowling (38) as assistance provided by the school administrators for teachers in the fields of teaching methods innovation, student management, teaching environment improvement, and so on. This support has been highlighted as a strong predictor of teachers’ job satisfaction and retention willingness (16, 39). Using a cross-lagged panel model and hierarchical linear modeling, Chen et al. (11) demonstrated that increased administrative support significantly alleviated teachers’ PNT during online teaching. Conversely, when teachers feel pressured rather than supported by administrators, they experience thwarted basic needs, leading to increased exhaustion and negative interactions with students (40).

While the associations between PNT, PSU, psychological distress, and perceived administrative support have been individually studied, few investigations have addressed these factors collectively. Integrating these factors with PNT in a single study focusing on teachers can enhance our understanding of their mental health, paving the way for more effective administrative support. This research adopts a longitudinal approach, allowing for between-group mean comparison. This methodology offers an advantage over previous cross-sectional studies (41, 42) by enabling the observation of trends in the effects of PNT on teachers’ psychological distress, PSU, and perceived administrative support across different groups. Through detailed analysis of teachers’ PNT status as well as its association with the three mentioned factors, the present study can provide valuable guidance for developing interventions for each PNT profile to enhance the well-being of Chinese primary and middle school teachers.

Consequently, the primary research questions for the current study are:

- How many distinct teacher profiles can be identified based on PNT using LPA?
- Do these distinct teacher groups exhibit differences in problematic smartphone use, psychological distress, and perceived administrative support at different time points?

## 2 Method

### 2.1 Procedure and participants

In a provincial city in central China, we conducted a longitudinal study to investigate factors associated with primary and middle school teachers. The study included two observation points: Time 1, which took place from November 19 to November 21, 2021, and Time 2, which occurred from January 5 to January 16, 2022. The duration of the follow-up period was approximately 2 months. At the outset, during Time 1, an outbreak of COVID-19 necessitated the closure of all primary and middle schools within the city, prompting an immediate shift to online teaching. By Time 2, schools had recommenced in-person instruction, having been operational for approximately 2 weeks.

**Figure 1:**
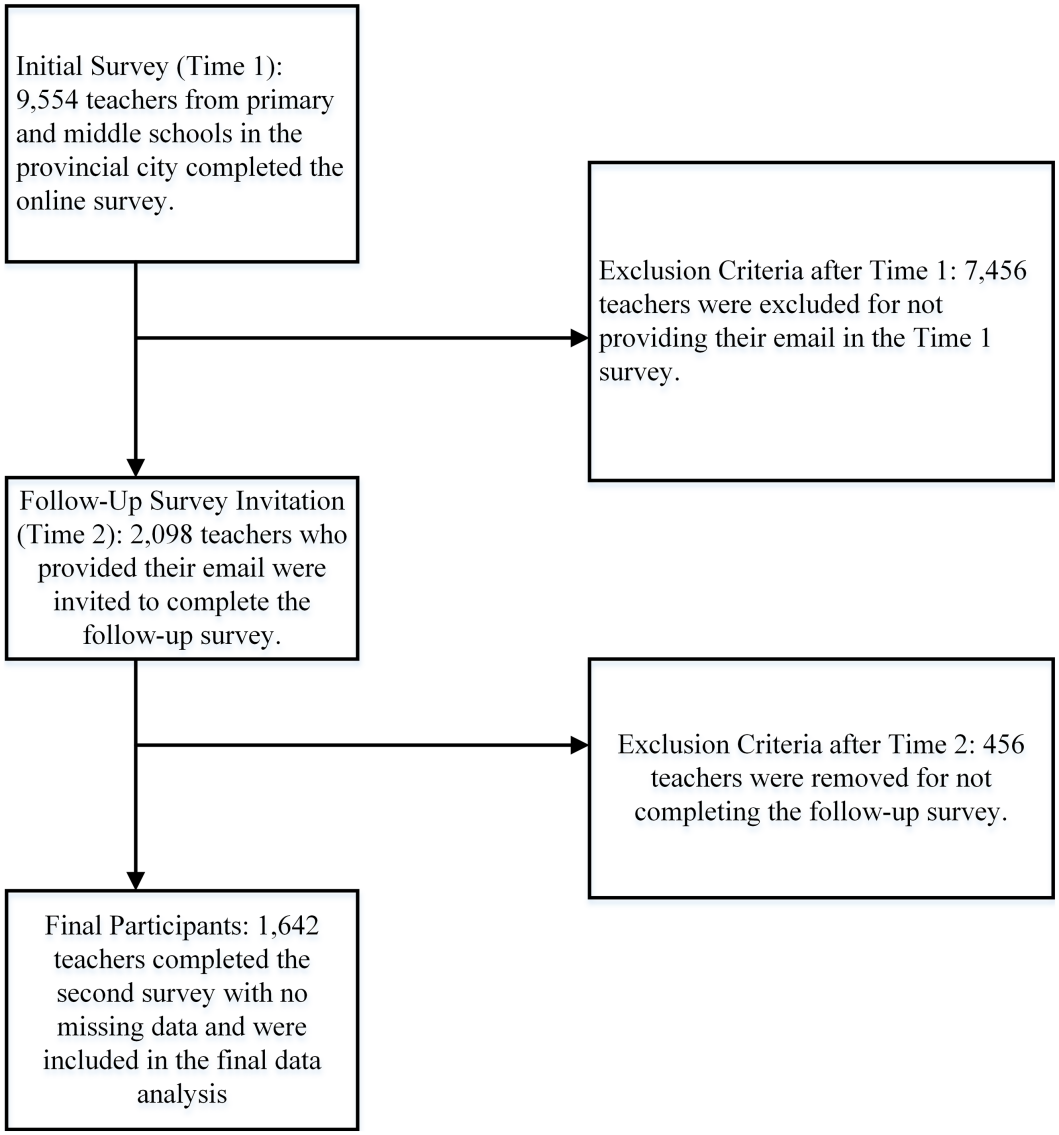
Flowchart depicting the sampling procedure and inclusion/exclusion criteria.

We employed a combination of convenience and purposive sampling methodologies to select participants. The inclusion and exclusion criteria, along with the participant screening procedure, are detailed in Figure 1. Specifically, we collaborated with the city’s Education Bureau to distribute an online questionnaire to primary and middle school principals, who then forwarded it to their teaching staff. Participation in the survey was entirely voluntary. The questionnaire included a query about teachers’ willingness to participate in a follow-up survey 2 months later, asking them to provide their email addresses if they were agreeable. Out of the initial 9,554 respondents, 2,098 teachers provided their email addresses and were subsequently sent the follow-up survey at Time 2. The inclusion criteria for our analysis required participants to provide a valid email address and complete the second survey. Of the teachers contacted for the follow-up, 1,642 responded, resulting in an attrition rate of 21.7%. We further confirmed that there was no obvious attrition bias, as evidenced by the non-significant differences between the datasets of 2,098 and 1,642 participants (*t* = 1.85, *p* = 0.06 for age; χ^2^ = 0.43, 1.72, and 0.15, *p* = 0.51, 0.19, and 0.70 for sex, school level, and school type, respectively). Notably, since the survey was conducted using the Questionnaire Star platform, comprehensive data collection was ensured with no missing data.

Participant demographic details are elucidated in Table 1. A substantial 96.3% of the teachers (*n* = 1,581) are associated with public educational institutions, with 70.6% (*n* = 1,159) engaged in primary education. The cohort predominantly comprises females, accounting for 79% (*n* = 1,305). Geographically, the majority of these teachers are based in counties (37.1%) and villages (55.7%). In terms of teaching tenure, there’s a discernible decline in the 16–20 years range, but other experience intervals exhibit a relatively even distribution. Compared to the demographic characteristics of the overall population of middle and primary school teachers in China (43), the participants in our study were, on average, approximately 3 years younger (*t* = −15.45, Cohen’s *d* = 0.38, indicating a small effect). The proportion of female teachers in our sample was higher (*χ*^2^ = 70.21, Cohen’s *W* = 0.21, representing a small effect), and there was a greater representation of primary school teachers (*χ*^2^ = 30.90, Cohen’s *W* = 0.14, also a small effect). Additionally, our sample included a higher percentage of public school teachers (*χ*^2^ = 5.71, Cohen’s *W* = 0.06, a trivial effect). Despite these statistically significant differences when compared with the population, the effect sizes were small to trivial. Therefore, it can be concluded that our study does not exhibit substantial sample bias in terms of sample composition.

**Table 1:** Participant characteristics (*n* = 1,642).

	*n* (%)
School type	
Public school	1,581 (96.3)
Private school	61 (3.7)
Level	
Middle school	483 (29.4)
Primary school	1,159 (70.6)
School location	
City	119 (7.2)
County	609 (37.1)
Village	914 (55.7)
Sex	
Male	337 (20.5)
Female	1,305 (79.5%)
Working years	
Under 5 years	465 (28.3)
6 years to 10	375 (22.8)
11 years to 15 years	275 (16.7)
16 years to 20 years	160 (9.7)
Over 21 years	367 (22.4)

Prior to the survey, participants were presented with an electronic informed consent form detailing the study’s objectives. This research secured ethical clearance from the Jiangxi Psychological Consultant Association (IRB ref.: JXSXL-2020-J013).

### 2.2 Instruments

In this study, we measured variables such as teachers’ PNT, problematic smartphone use, psychological distress, and perceived administrative support using the instruments described below. Both the Chinese and English versions of each instrument can be found in Supplementary Table S1. We also collected background information, including school level, type, location, years of employment, and gender. Although PNT was also measured in the follow-up survey, this study exclusively utilized the Time 1 data. The Time 2 data, gathered for measurement invariance in a different study (20), were not relevant or used in the present research. All other variables were assessed at both times. Details regarding the individual validity and reliability of each measure employed in this study will be presented later. The discussion of the overall validity, considering all instruments collectively, will be specifically addressed in the results section.

#### 2.2.1 Psychological need thwarting scale of online teaching

We used the Psychological Need Thwarting Scale of Online Teaching (PNTSOT) to measure the extent to which teachers’ psychological needs were thwarted (8). Designed based on SDT, the PNTSOT assesses three core psychological needs: autonomy, competence, and relatedness. The scale comprises three subscales, each having seven items. Teachers were instructed to reflect on the situation when emergency online teaching was initiated. Examples include: “I feel compelled to follow a predetermined online teaching method during the pandemic” (for autonomy thwarting), “Online teaching during the pandemic occasionally makes me feel inadequate” (for competence thwarting), and “While teaching online during the pandemic, I often feel disconnected from my colleagues and supervisors” (for relatedness thwarting). Respondents rated these items on a seven-point Likert scale, with higher scores indicating a stronger perception of their psychological needs being thwarted in an online teaching context. The PNTSOT’s three-factor structure was validated by previous studies (8, 11). In our research, we conducted an evaluation of the higher-order factor structure of the scale, incorporating three distinct types of PNT as first-order factors. The confirmatory factor analysis (CFA) yielded results that affirm the factorial validity of the scale. The analysis demonstrated a chi-square (*χ*^2^) value of 509.885 with 50 degrees of freedom. The Comparative Fit Index (CFI) was recorded at 0.968, and the Non-Normed Fit Index (NNFI) stood at 0.958, both indices indicating a robust model fit. The Root Mean Square Error of Approximation (RMSEA) was observed at 0.075, while the Standardized Root Mean Square Residual (SRMR) was calculated to be 0.066. These values collectively suggest a satisfactory fit of the model. Furthermore, the internal consistency of the PNTSOT was found to be commendable, with ordinal McDonald’s ω values for the three subscales being 0.84, 0.88, and 0.95, respectively, indicating a high level of reliability.

#### 2.2.2 Smartphone application-based addiction scale

To evaluate teachers’ problematic smartphone use, we employed the Smartphone Application-Based Addiction Scale (SABAS) (44). The SABAS consists of six items, each representing a criterion from the addiction components model. This scale identifies the risk of addiction to smartphone applications. Items are rated on a six-point Likert scale, from 1 (strongly disagree) to 6 (strongly agree). Higher scores indicate a higher risk of addiction. The scale was translated into simplified Chinese and demonstrated strong factorial validity and impressive internal consistency with a coefficient of 0.81 (45). SABAS also remained consistent over a three-month period (45). In our study, the SABAS demonstrated a unidimensional structure. CFA indicated that the structure had a satisfactory model fit. At Time 1, the *χ*^2^ value was 51.275 with 9 degrees of freedom, the CFI was 0.991, the NNFI was 0.985, the RMSEA was 0.054, and the SRMR was 0.037. At Time 2, the chi-square (*χ*^2^) value was 47.607 with 9 degrees of freedom, the CFI was 0.994, the NNFI was 0.990, the RMSEA was 0.051, and the SRMR was 0.037. The internal consistency of the SABAS was commendable, with ordinal McDonald’s ω values of 0.91 at Time 1 and 0.93 at Time 2, respectively. At Time 1, participants were prompted to reflect on their smartphone usage during the onset of emergency online teaching. Later, they were asked to describe their phone use from the recent months.

#### 2.2.3 The depression, anxiety, and stress Scale-21 version

In our current study, we employed the Depression, Anxiety, and Stress Scale-21 Version (DASS-21) (46) to assess psychological distress among teachers. This scale, comprising 21 items evenly distributed across three subscales—depression, anxiety, and stress—is extensively used for evaluating distinct negative emotional states. However, recent research (47, 48) suggests that it primarily measures overall psychological distress. We interpreted the mean scores of the DASS-21 subscales as indicators of teachers’ psychological distress. Participants rated their feelings on a four-point Likert scale, from 0 (never) to 3 (almost always), with higher scores indicating greater distress. At Time 1, participants were asked to reflect on their experiences during the initiation of emergency online teaching, while at Time 2, they recalled their feelings from the previous 2 weeks.

Building upon our previous study (49), we found that the DASS-21 demonstrated acceptable factorial validity in its original three-factor structure. The CFA from our earlier research yielded *χ*^2^ (df) values of 1373.082 (186) and 1226.004 (186), CFI values of 0.992 and 0.994, NNFI values of 0.991 and 0.994, RMSEA values of 0.062 and 0.058, and SRMR values of 0.044 and 0.038 at Time 1 and Time 2, respectively. Furthermore, our prior research confirmed the DASS-21’s acceptable internal and test–retest reliability among Chinese teachers, with all three subscales exhibiting Cronbach’s α values exceeding 0.85 and intraclass correlation coefficients above 0.70. The time-invariance feature of the DASS-21 was also established in our earlier work. In the current study, the McDonald’s ω for the depression, anxiety, and stress subscales were 0.892, 0.866, and 0.865 at Time 1, and 0.916, 0.890, and 0.901 at Time 2, respectively.

#### 2.2.4 Teachers’ perception of administrators’ support scale

In this study, we utilized the Teachers’ Perception of Administrators’ Support Scale (TASS), developed by Chen et al. (11), to evaluate teachers’ perceptions of administrative support during the shift to emergency online teaching amid the COVID-19 pandemic. The TASS, an adaptation of the Scale of Technology Users’ Beliefs, measures expectations of key stakeholders in the context of technology use (50). At Time 1, participants were asked to reflect on the immediate context of emergency online teaching, whereas at Time 2, they were prompted to recall their experiences from 2 months prior, marking the onset of emergency online teaching. The TASS comprises four items, with examples including: ‘Administrators expect teachers to transition smoothly to online teaching during the outbreak’ and ‘School administrators have provided the majority of essential resources to support teachers’ shift to online instruction during the pandemic’. Responses were elicited on a five-point Likert scale, ranging from 1 (strongly disagree) to 5 (strongly agree). In our study, we tested its factorial validity within a one-factor structure. The CFA results showed *χ*^2^ (df) values of 12.552 (2) and 3.127 (2), CFI values of 0.993 and 0.999, NNFI values of 0.980 and 0.991, RMSEA values of 0.057 and 0.019, and SRMR values of 0.034 and 0.018 at Time 1 and Time 2, respectively. The ordinal McDonald’s ω coefficient for TASS was 0.92 at Time 1 and 0.93 at Time 2, indicating high internal consistency.

### 2.3 Data analysis

To analyze the temporal trends in teachers’ psychological distress, perceived administrative support, and PSU over two distinct time points, ensuring the temporal reliability of the instruments used is essential. This verification is key to effectively identifying potential measurement bias in the longitudinal framework. For a comprehensive assessment of this test–retest reliability, the Intraclass Correlation Coefficient (ICC) was employed. This robust statistical method assesses the consistency of continuous measurements across time by evaluating both correlation and agreement between two measurement sets. The ICC, ranging from 0 (no reliability) to 1 (perfect reliability), compares the variability of scores between subjects against the total variation across all measurements and subjects. We will adopt a two-way mixed-effects model, suitable for repeated measurements on the same subjects, to calculate the ICC, thereby accounting for both systematic and random errors. An ICC value above 0.70, as recommended by Thompson et al. (51), is considered indicative of good reliability.

Subsequently, we conducted confirmatory factor analyzes (CFA) on our data collected at Time 1 and Time 2 to establish the construct validity of our metrics, focusing on both factorial and convergent validity. For evaluating factorial validity, we utilized several specific fit indices in our CFA. We determined that both the Comparative Fit Index (CFI) and the Tucker-Lewis Index (TLI), also known as the Non-Normed Fit Index (NNFI), should exceed the threshold of 0.90 to indicate a good model fit. Additionally, we aimed for the Root Mean Square Error of Approximation (RMSEA) to be below 0.06 and the Standardized Root Mean Square Residual (SRMR) to be under 0.08, as these values are indicative of an acceptable model fit according to the standards set by Hu and Bentler (52). Furthermore, to assess convergent validity through our CFA, we calculated the Composite Construct Reliability (CCR) and the Average Variance Extracted (AVE) for each construct. These metrics are crucial for determining the extent to which a set of items represents a single latent construct. We adhered to the guidelines provided by Fornell & Larcker (53) and Hair et al. (54), confirming convergent validity when the CCR exceeds 0.70 and the AVE is above 0.50 for each construct. These thresholds ensure that our constructs are reliable and that a significant portion of the variance in the observed variables is accounted for by the latent construct.

After validating the integrity and quality of our measurement instruments, we began a preliminary examination. This initial phase involved analyzing descriptive statistics, paired *t*-tests, and zero-order correlations of the observed variables, using their raw scores without any transformations.

Subsequently, employing the tidyLPA package in R, we conducted Latent Profile Analysis (LPA). This methodological approach was specifically applied to identify the number of latent profiles within the three dimensions of online teaching PNT, based on the 12-item PNTSOT scale. LPA, as emphasized by Tein et al. (55), is a statistical technique used to identify distinct subgroups within a heterogeneous dataset based on observed variables. LPA classifies individuals into mutually exclusive groups, or ‘profiles’, based on their response patterns. This method is particularly useful in uncovering hidden structures within complex data, allowing for a more nuanced understanding of the underlying patterns and relationships. By applying LPA in our study, we aimed to discern and characterize consistent latent groups, thereby enhancing the depth and precision of our data analysis. To determine the optimal number of profiles, we considered metrics such as Akaike’s Information Criterion (AIC), Bayesian Information Criterion (BIC), the Sample-Adjusted BIC (SABIC), Integrated Complete-data Likelihood (ICL), Entropy, and the Bootstrap Likelihood Ratio Test (BLRT). A better model fit is typically indicated by lower values of AIC, BIC, and SABIC, along with higher values of ICL and entropy, the latter ideally being higher than 0.90 (56). The BLRT (Bootstrap Likelihood Ratio Test) comparisons were also integral to our analysis, aiding in the evaluation of model structures with ‘K’ and ‘K-1’ classes. Consistent with the principle of model parsimony, a model with ‘K’ classes is considered redundant if the BLRT does not demonstrate significant improvements over the ‘K-1’ class model. This lack of significant improvement implies that the additional category in the ‘K’ class model fails to provide substantial new information. Moreover, in light of the larger sample size of our study, we adopted the ‘elbow-criterion’ as described by Morin and Wang (57). This method entails selecting a profile solution at the juncture where the curve begins to plateau, thereby indicating an optimal balance between model complexity and explanatory power.

In this study, after identifying potential latent profiles, our primary aim was to explore how these profiles differ in their perceptions of administrative support, psychological distress, and patterns of PSU across two survey time points. To achieve this, Structural Equation Modeling (SEM) was utilized. This technique was instrumental in assessing the varying impacts that distinct latent classes had on their corresponding latent variables: administrative support, psychological distress, and PSU, both at the initial survey (Time 1) and the follow-up (Time 2). The indicators used for these variables were items from the TASS for administrative support, the SABAS for PSU, and the mean scores of the three emotional disorder subscales of the DASS-21. In the SEM framework, we accounted for sex, age, and autoregressive effects by linking each latent variable at time 1 with its corresponding variable at time 2, thereby improving the precision of our estimations. This approach also allowed for the simultaneous estimation of the effects of latent classes on the latent variables, a strategy that helped in mitigating the risk of inflating the Type I error rate. It’s crucial to note that our analysis did not incorporate cross-lagged effects, as the study’s scope did not include investigating the causal relationships among administrative support, psychological distress, and PSU. In the SEM section, we first evaluated the overall model fit using the criteria established in the CFA mentioned previously. Following this, the path coefficients were rigorously examined and duly reported.

Before presenting our results, we conducted a thorough examination of the underlying assumptions necessary for conducting LPA and SEM, with a particular focus on the normality distribution of the indicators for the latent classes or variables (56, 58). For each item of the selected scale, we observed that skewness ranged between −0.69 and 1.06, while kurtosis varied between −1.05 and 1.26. Regarding the mean score of the subscale of the DASS-21, which serves as an indicator for psychological distress, skewness values ranged from 1.12 to 1.89, and kurtosis values were between 1.05 and 4.38. These values align with Kline’s criteria (58), which state that absolute skewness higher than 3.0 and absolute kurtosis above 8 are indicative of severe skewness and kurtosis, respectively. Furthermore, we employed the Intraclass Correlation Coefficient (ICC) to test the assumption of independence, specifically using 12 items of PNTSOT. We found that small ICC values, with the highest being 0.022, suggest a negligible nested effect. This finding allows us to consider the participants as independently sampled, which is also a critical assumption for Latent Profile Analysis (LPA) (59).

## 3 Results

### 3.1 Test–retest reliability and construct validity

In an evaluation of test–retest reliability, ICCs were initially utilized to compare observed means across two distinct time points. The findings revealed that two of the three instruments exhibited commendable reliability over time, with both psychological distress and PSU yielding an ICC of 0.74. However, TASS displayed a slightly lower ICC of 0.62.

**Figure 2:**
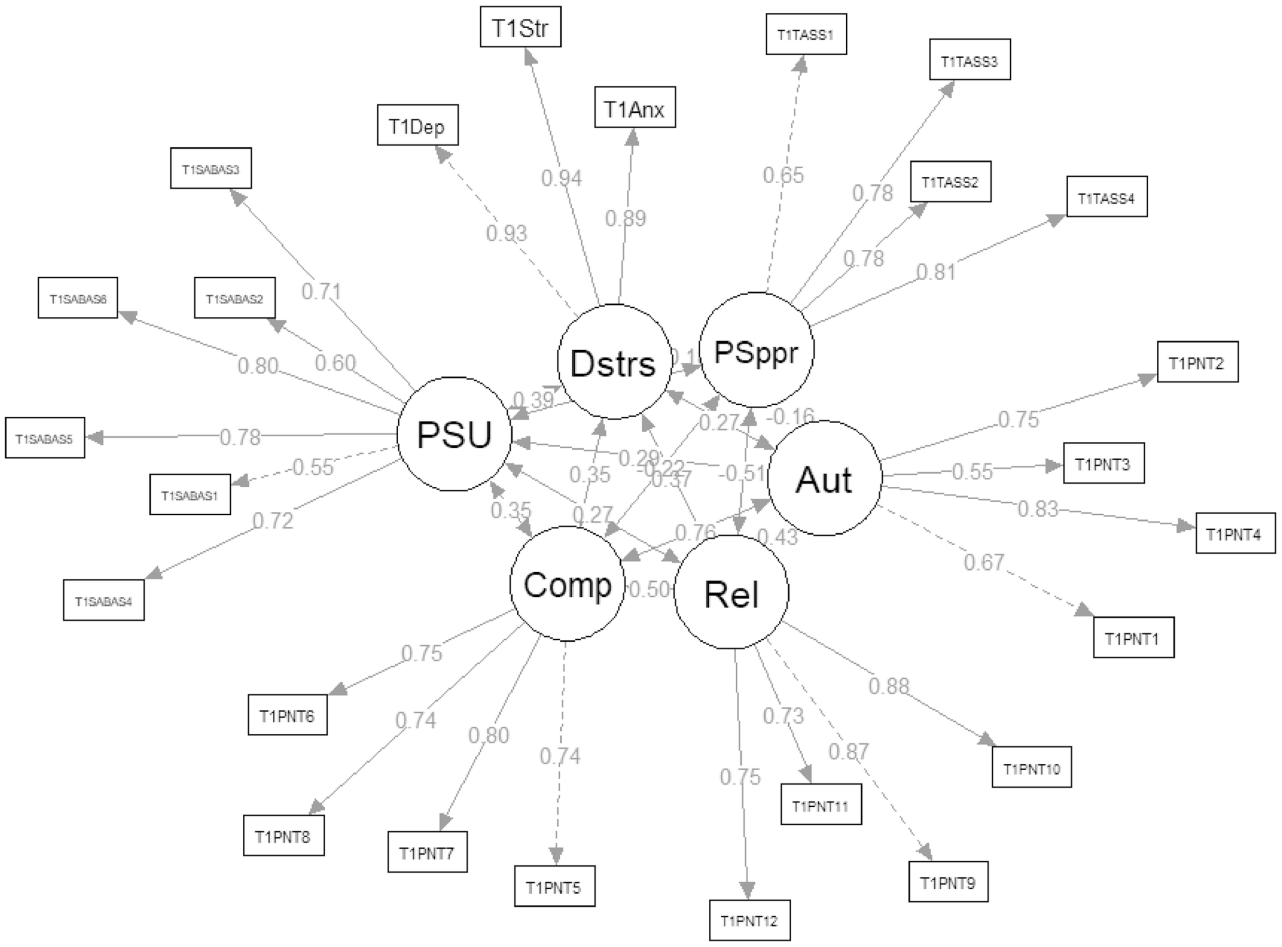
Measurement model at time 1. PSU: problematic smartphone use; Dstrs: psychological distress; PSppr: perceived administrative support; Comp: competence thwarting; Aut: autonomy thwarting; Rel: relatedness thwarting.

**Figure 3:**
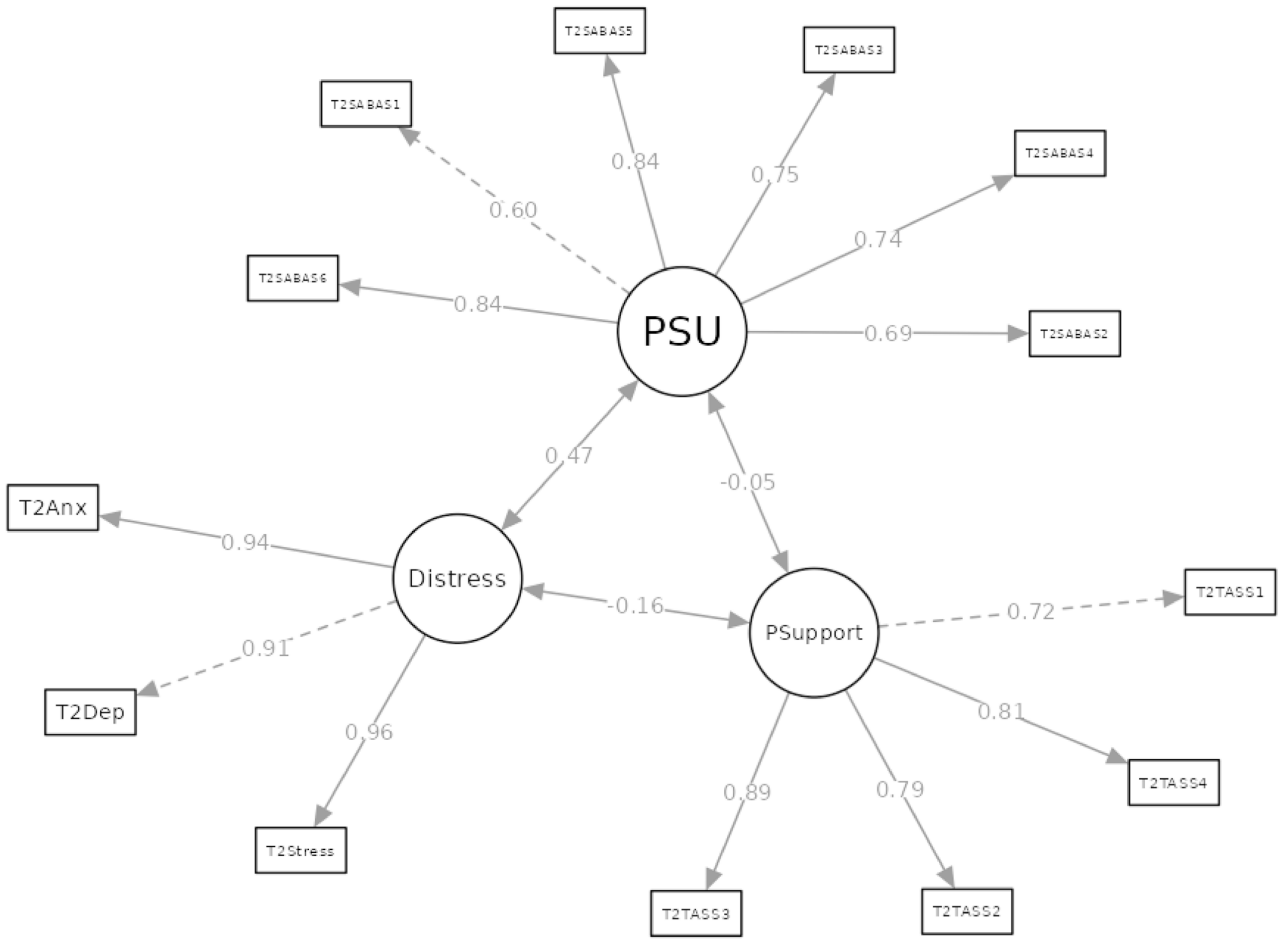
Measurement model at time 2. PSU: problematic smartphone use; Distress: psychological distress; PSupport: perceived administrative support.

Subsequent CFA for the measurement models at both time points further substantiated acceptable construct validity. Model fit indices presented in Table 2 endorsed the factorial validity of the instruments. Factor loadings for each item, illustrated in Figures 2, 3, consistently exceeded the threshold of 0.50. Based on these loadings, the Average Variance Extracted (AVE) and Composite Reliability (CR) were computed for each latent variable. For Time 1, the AVE and CR values were as follows: PSU (0.49, 0.85), psychological distress (0.85, 0.94), autonomy thwarting (0.50, 0.80), competence thwarting (0.57, 0.84), relatedness thwarting (0.66, 0.88), and perceived administrative support (0.57, 0.84). For Time 2, the AVE and CR for PSU were 0.56 and 0.88 respectively, with psychological distress at 0.88 and 0.96, and perceived administrative support at 0.65 and 0.88. Taken collectively, the aforementioned results substantiate that the observed scores derived from this study exhibit consistent temporal reliability and demonstrate commendable construct validity, indicating no obvious longitudinal measurement bias.

**Table 2:** Model fit for measurement models for all variables in time 1 and time 2.

	*χ*^2^ (df)	CFI	NNFI	RMSEA	SRMR
Time 1	1207.98 (142)	0.953	0.944	0.068	0.068
Time 2	259.22 (62)	0.984	0.980	0.044	0.043

CFI, comparative fit index; NNFI, non-normed fit index; RMSEA, root mean square error of approximation; SRMR, standardized root mean square residual.

### 3.2 Preliminary analysis

Table 3 displays the mean observed scores (with standard deviations) for the study’s variables and their associations. Notably, among the three types of need thwarting, autonomy and competence thwarting were more prominent, both exceeding relatedness thwarting. Additionally, the scores for PSU, psychological distress, and perceived administrative support at Time 2 were all lower than at Time 1. Paired *t*-tests were further conducted, revealing that PSU and perceived administrative support were significantly lower at Time 2 compared with Time 1 (PSU: *t* = 7.81, *p* < 0.01; perceived administrative support: *t* = 5.81, *p* < 0.01), while there was no significant difference in psychological distress between the two time points.

**Table 3:** Descriptive statistics and Pearson correlations among internet activities, three kinds of psychological needs thwarting, perceived administrative support, distress, and problematic smartphone use.

	*M* (SD)	1	2	3	4	5	6	7	8
1. Autonomy thwarting	15.69 (4.77)	1							
2. Competence thwarting	15.73 (5.12)	0.62^**^	1						
3. Relatedness thwarting	9.57 (4.29)	0.36^**^	0.44^**^	1					
4. Perceived administrative support_Time 1	15.19 (2.65)	−0.13^**^	−0.18^**^	−0.44^**^	1				
5. Perceived administrative support_Time 2	14.78 (2.77)	−0.13^**^	−0.14^**^	−0.31^**^	0.45^**^	1			
6. Distress _Time 1	9.81 (10.10)	0.23^**^	0.31^**^	0.34^**^	−0.16^**^	−0.12^**^	1		
7. Distress _Time 2	9.56 (10.89)	0.17^**^	0.22^**^	0.26^**^	−0.15^**^	−0.14^**^	0.58^**^	1	
8. Problematic smartphone use_Time 1	17.52 (5.93)	0.24^**^	0.30^**^	0.23^**^	−0.08^**^	^.^-0.07^**^	0.35^**^	0.31^**^	1
9. Problematic smartphone use_Time 2	16.46 (6.15)	0.17^**^	0.22^**^	0.17^**^	−0.09^**^	−0.03	0.29^**^	0.43^**^	0.59^**^

^**^p < 0.01.

In terms of variable correlations, we observed significant positive correlations among the three types of psychological needs thwarting: autonomy, competence, and relatedness. The correlation coefficients for these variables were as follows: between autonomy and competence, *r* = 0.62 (*p* < 0.01), between autonomy and relatedness, *r* = 0.36 (*p* < 0.01), and between competence and relatedness, *r* = 0.44 (*p* < 0.01). These coefficients indicate a moderate to strong positive relationship among these types of needs thwarting, suggesting that they often occur concurrently within our sample.

Furthermore, we found a negative correlation between psychological needs thwarting and perceived administrative support at both measured time points. The coefficients ranged from −0.13 to −0.44 (all *p* < 0.01) at Time 1 and from −0.13 to −0.31 (all *p* < 0.01) at Time 2, indicating a consistent moderate negative relationship. This suggests that higher levels of psychological needs thwarting are associated with lower levels of perceived administrative support.

Lastly, positive correlations were observed between psychological needs thwarting and both psychological distress and problematic smartphone use. The correlation with psychological distress ranged from 0.17 to 0.34 (all *p* < 0.01), and with problematic smartphone use, it ranged from 0.17 to 0.30 (all *p* < 0.01) across two time points. These moderate positive correlations suggest a notable relationship between the thwarting of psychological needs and increased levels of psychological distress and problematic smartphone use. It’s worth noting that the correlations between the PNTs at Time 1 and other Time 1 variables were stronger than those with Time 2 variables.

### 3.3 Latent profile analysis

**Figure 4:**
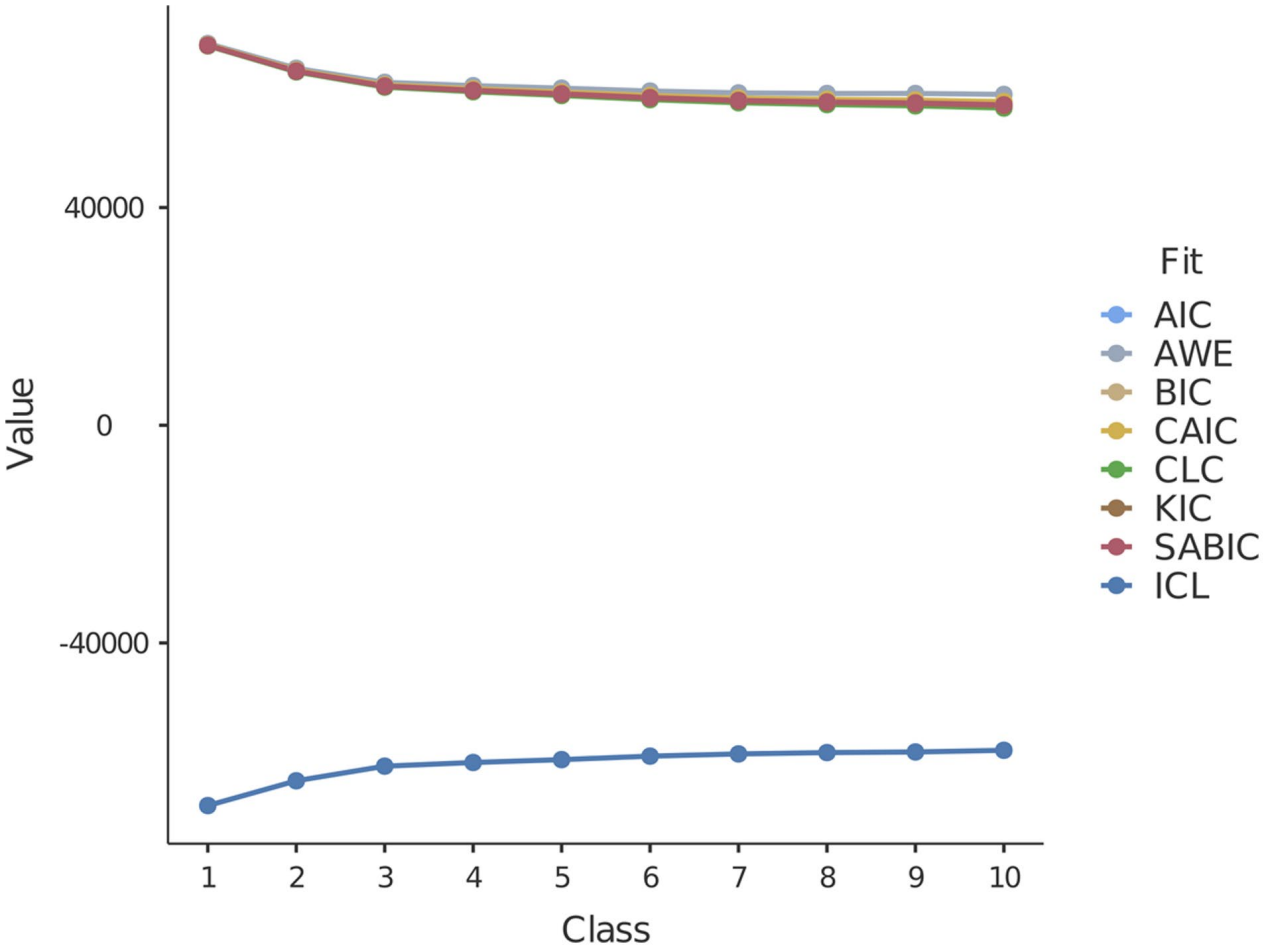
Elbow plot illustrating information criteria values for all latent profiles. Take note that a 3-class profile was selected.

LPA was conducted to identify distinct latent profiles within our sample. The fit information for 10 potential profiles is presented in Table 4. Based on indicators such as AIC, BIC, SABIC, and ICL, we observed that as the number of classes increased, these values continuously decreased (except for ICL, which increased), and the *value of p*s of the BLRT was significant for each profile. A significant BLRT result indicates that the model with more profiles represents the data better than the model with fewer profiles. Given the complexity of determining the optimal number of categories based solely on these indicators, we employed a strategy focusing on the disparity in AIC, BIC, SABIC, and ICL values among successive profiles. Our objective was to identify significant changes in value within these nested models. This analysis highlighted that the 3-profile model exhibited the most substantial variation in ΔAIC, ΔBIC, ΔSABIC, and ΔICL values compared to the preceding profile. An examination of Figure 4, which illustrates the ‘elbow’ in the plot, further supports the distinctiveness of the 3-profile model. Consequently, the three-profile solution was deemed optimal, balancing model performance and simplicity. Notably, the 3-profile model was the only one with a value exceeding 0.90.

**Table 4:** Summary of the model selection for the latent profiles based on three kinds of psychological needs thwarting.

Class	LogLik	AIC	Δ AIC	BIC	ΔBIC	SABIC	Δ SABIC	ICL	Δ ICL	BLRT(*p*)	Entropy
1-Profile	−34853.18	69754.36		69884.05		69807.80		−69884.05			1.00
2-Profile	−32460.97	64995.94	−4758.42	65195.87	−4688.17	65078.33	−4729.47	−65325.97	4558.07	0.010	0.89
3-Profile	−31057.89	62215.77	−2780.17	62485.96	−2709.92	62327.11	−2751.22	−62639.36	2686.62	0.010	0.91
4-Profile	−30637.28	61400.57	−815.20	61741.00	−744.96	61540.86	−786.26	−61966.12	673.24	0.010	0.90
5-Profile	−30289.88	60731.75	−668.82	61142.43	−598.57	60900.99	−639.87	−61438.16	527.97	0.010	0.88
6-Profile	−29909.31	59996.62	−735.13	60477.55	−664.88	60194.81	−706.18	−60798.19	639.97	0.010	0.89
7-Profile	−29625.48	59454.95	−541.67	60006.13	−471.42	59682.09	−512.72	−60391.43	406.76	0.009	0.88
8-Profile	−29454.88	59139.76	−315.19	59761.18	−244.95	59395.85	−286.24	−60143.66	247.76	0.01	0.88
9-Profile	−29340.20	58936.40	−203.36	59628.07	−133.11	59221.44	−174.41	−60034.03	109.64	0.010	0.88
10-Profile	−29137.78	58557.56	−378.85	59319.48	−308.60	58871.54	−349.90	−59724.00	310.02	0.010	0.88

LogLik refers to the model’s log-likelihood, Akaike’s Information Criterion (AIC), Bayesian Information Criterion (BIC), the Sample-Adjusted BIC (SABIC), Integrated Complete-data Likelihood (ICL), and the Bootstrap Likelihood Ratio Test (BLRT). The model marked in bold is the most optimized, selected based on its statistical analysis and clarity in interpretation.

**Figure 5:**
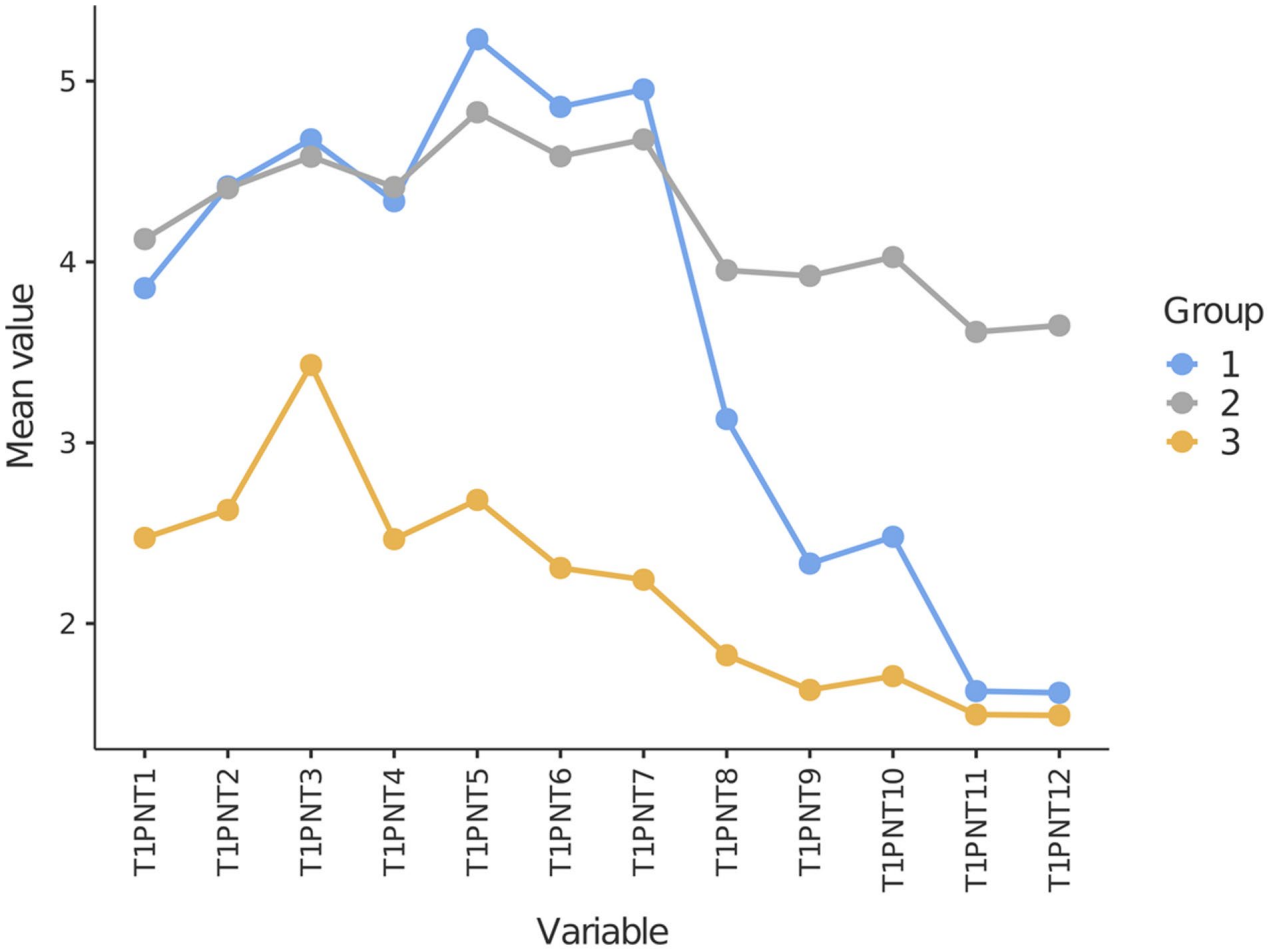
Line graph depicting profile comparisons for psychological needs thwarting.

Next, we took a closer look at the characteristics of these three profiles. Specifically, each of the identified profiles is characterized by distinct mean scores across three types of psychological needs thwarting, as detailed in Table 5. Figure 5 provides a graphical representation of the individual item scores within the PNTSOT. Our findings indicate significant differences in mean scores for autonomy, competence, and relatedness thwarting across the groups, with *F*-values ranging from 436.88 to 1784.43 (all *p* < 0.01) and large effect sizes (η^2^ of 0.35, 0.61, and 0.69, respectively). In exploring autonomy and competence thwarting, the Games-Howell post-hoc test revealed that Class 1 and Class 2 exhibited significantly higher levels than Class 3. For relatedness thwarting, the groups were ranked from highest to lowest as follows: Class 2, Class 1, and Class 3.

**Table 5:** The comparisons between three -profiles in terms of three kinds of psychological needs thwarting.

	Class 1 (*n* = 757, 46.1%)	Class 2 (*n* = 452, 27.5%)	Class 3 (*n* = 433, 26.4%)	*F*-test (*value of p*)	*Post-hoc*
Variable: Mean (SD)					
1. Autonomy thwarting	17.29 (3.93)	17.53 (3.37)	10.99 (4.19)	436.88 (<0.01)	C1 > C3; C2 > C3
2. Competence thwarting	18.17 (3.09)	18.04 (3.50)	9.06 (3.09)	1273.26 (<0.01)	C1 > C3; C2 > C3
3. Relatedness thwarting	8.05 (2.48)	15.21 (2.48)	6.33 (2.17)	1784.43 (<0.01)	C2 > C1 > C3
4. Perceived administrative support_Time 1	15.57 (2.28)	13.62 (2.14)	16.18 (3.01)	136.31 (<0.001)	C3 > C1 > C2
5. Perceived administrative support_Time 2	14.93 (2.55)	13.74 (2.49)	15.62 (3.08)	56.48 (<0.001)	C3 > C1 > C2
6. Psychological distress _Time 1	9.61 (8.86)	13.96 (12.03)	5.84 (8.10)	78.60 (<0.01)	C2 > C1 > C3
7. Psychological distress _Time 2	9.34 (9.90)	13.09 (12.83)	6.27 (9.10)	46.14 (<0.01)	C2 > C1 > C3
8. Problematic smartphone use_Time 1	18.22 (5.63)	18.71 (5.80)	15.07 (5.90)	54.31 (<0.01)	C1 > C3; C2 > C3
9. Problematic smartphone use_Time 2	16.99 (5.95)	17.39 (6.14)	14.57 (6.12)	29.39 (<0.01)	C1 > C3; C2 > C3

Post hoc comparisons were performed using the Games-Howell test, which is appropriate for datasets with unequal variances and different sample sizes.

Based on the unique characteristics of each class, we labeled these and reported their prevalence within our sample. Class 1, labeled as ‘High Autonomy-High Competence and Moderate Relatedness Thwarting,’ comprises the largest group at 46.1% (757 participants). This indicates a substantial segment with high thwarting in both autonomy and competence (evidenced by scores above the median in these subscales) and moderate levels of relatedness thwarting, positioned between Class 2 and Class 3. Class 2, ‘High Autonomy-High Competence and High Relatedness Thwarting,’ encompasses 27.5% of the sample (452 individuals) and mirrors Class 1 in autonomy and competence but exhibits markedly higher relatedness thwarting. Finally, Class 3, ‘Low Psychological Needs Thwarting,’ accounts for 26.4% of the sample (433 participants) and is characterized by consistently scoring below the median across all PNTSOT dimensions. This class’s lower level of needs thwarting across all areas highlights a contrastingly positive psychological experience compared to the other two classes. The distribution of these classes underscores the diversity of psychological needs thwarting experiences, with each class reflecting a distinct interplay of autonomy, competence, and relatedness thwarting in our sample.

Furthermore, in addressing potential sources of bias in our LPA, we considered factors such as sample representativeness, measurement accuracy, and model selection criteria. The composition of the sample was carefully examined to ensure a broad representation of the population of interest (for more details, please refer to the Procedure and Participants’ section). To enhance measurement accuracy, we utilized validated scales, specifically the PNTSOT, and took measures to ensure data quality. The construct validity of the PNTSOT, as mentioned earlier, was also verified (for further information, please see the Test–Retest Reliability and Construct Validity section). Our model selection process was guided not only by statistical fit indices but also by the theoretical coherence and interpretability of the profiles. These measures were implemented to mitigate potential biases and thus enhance the reliability of our findings.

### 3.4 Structural equation modeling: delineating the differences across distinct latent classes

**Figure 6:**
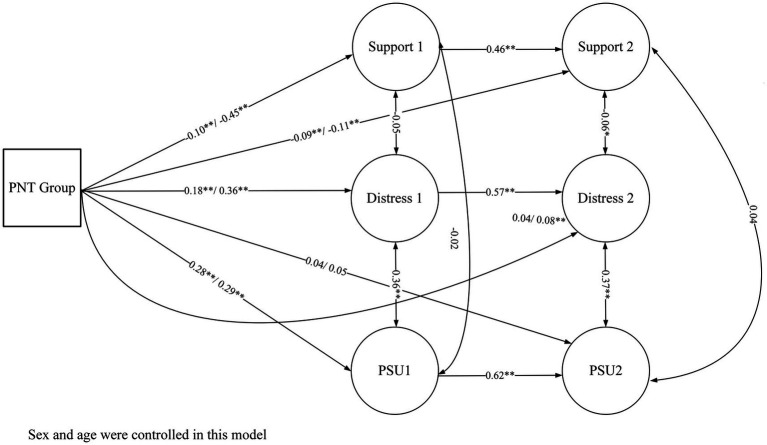
Structural equation modeling analysis of latent variables in distinct PNT groups over time. PNT Group: Psychological need thwarting latent classes; Support 1 and Support 2 represent administrative support at Time 1 and Time 2, respectively. Distress 1 and Distress 2 denote psychological distress at Time 1 and Time 2, respectively. PSU 1 and PSU 2 indicate problematic smartphone use at Time 1 and Time 2, respectively. ** *p* < 0.01, * *p* < 0.05. The figure presented above utilizes Class 3 as the reference group. To maintain brevity and clarity of presentation, results using Class 1 as the reference group are not depicted separately within the figure.

Using Maximum Likelihood (ML) estimation and controlling for sex and age, the model’s fit was confirmed, as evidenced by the following metrics: *χ*^2^ (df) = 2853.54 (370), Comparative Fit Index (CFI) = 0.917, Non-Normed Fit Index (NNFI) = 0.903, Root Mean Square Error of Approximation (RMSEA) = 0.065, and Standardized Root Mean Square Residual (SRMR) = 0.049. The path coefficients for the two dummy variables were then scrutinized, with Class 3 serving as the reference, as illustrated in Figure 6. At the initial assessment (time 1), Classes 1 and 2, compared to Class 3, were found to have significantly lower levels of perceived administrative support (Dummy 1: Class 1 vs. Class 3, *β* = −0.10, *t* = −3.40, *p* < 0.01; Dummy 2: Class 2 vs. Class 3, *β* = −0.45, *t* = −14.08, *p* < 0.01) and increased distress (Dummy 1: Class 1 vs. Class 3, *β* = 0.18, *t* = 6.00, *p* < 0.01; Dummy 2: Class 2 vs. Class 3, *β* = 0.36, *t* = 12.04, *p* < 0.01), along with elevated PSU (Dummy 1: Class 1 vs. Class 3, *β* = 0.28, *t* = 8.28, *p* < 0.01; Dummy 2: Class 2 vs. Class 3, *β* = 0.29, *t* = 8.66, *p* < 0.01).

At the subsequent time point (time 2), although the differences between Class 3 and the other classes were reduced compared to the initial time frame, Class 3 still maintained significantly higher support (Dummy 1: Class 1 vs. Class 3, *β* = −0.09, *t* = −2.96, *p* < 0.01; Dummy 2: Class 2 vs. Class 3, *β* = −0.11, *t* = −3.52, *p* < 0.01) and lower distress compared to Class 2 (*β* = 0.08, *t* = 2.86, *p* < 0.01). Nonetheless, the variations in PSU between Class 3 and the other classes did not reach statistical significance.

Furthermore, the analysis was replicated with a shift in the reference category from Class 3 to Class 1 to enable a more direct comparison between Classes 1 and 2. The model’s fit indices indicated an excellent fit, with both the CFI and the NNFI surpassing the threshold of 0.90, and the RMSEA and the SRMR registering below 0.07. The path coefficient outcomes revealed that Class 1 reported higher levels of administrative support than Class 2 across the two measured time points (Time 1: *β* = −0.40, *t* = −14.40, *p* < 0.01; time 2: *β* = −0.06, *t* = −2.27, *p* = 0.02). Moreover, Class 2 exhibited higher levels of psychological distress than Class 1 at both Time 1 and Time 2 (Time 1: *β* = 0.26, *t* = 10.02, *p* < 0.01; Time 2: *β* = 0.06, *t* = 2.64, *p* < 0.01). In terms of PSU, Class 2’s scores were significantly greater than those of Class 1 at Time 1. (*β* = 0.13, *t* = 4.55, *p* < 0.01).

## 4 Discussion

Empirically, the three basic needs of thwarting have been shown to correlate, albeit not strongly. Moreover, individuals can experience varied PNT when faced with identical challenges. This variability gives rise to potential profiles. Consequently, this study primarily seeks to determine the number of unique groups that arise from teachers based on PNT. By adopting a person-centered approach – LPA, and comparing 10 latent profiles, it was determined that the 3-profile model is the most optimal, as evidenced by variations in AIC, BIC, SABIC, and ICL values. Given the close relationship between PNT and PSU, psychological distress, and perceived administrative support, this study also aims to examine the differences in their associations across the three distinct PNT profiles at two time points (Time 1 and Time 2). Detailed interpretations of these findings will be presented in the subsequent sections.

### 4.1 Psychological need thwarting profiles

In line with prior research (17), this study confirms that while the three PNT types are positively correlated, they exhibit unique characteristics. This distinction justified the use of LPA to segment the sample into different groups. Unlike the variable-centered approach, LPA enables researchers to determine if there are potential subgroups within a sample that share common characteristics (26). This method offers insights that complement the traditional variable-centered approach (60). As anticipated, distinct PNT profiles were identified among the teacher population. Upon comparison, only the 3-profile model was deemed optimal. Notable differences in the mean scores for the three PNT types were observed across these profiles. The first profile, Class 1, constitutes the majority (46.1%) of the sample and is characterized as “High autonomy, High competence, and Moderate relatedness thwarting.” Both autonomy and competence thwarting scores in this class exceed the median, while the relatedness score is relatively lower but still higher than that of Class 3. Class 2, representing 27.5% of the sample, is labeled “High autonomy, High competence, and High relatedness thwarting,” with no thwarting type scores falling below the median. In contrast, Class 3, which makes up 26.4% of the sample, is described as “Low psychological needs thwarting,” with all scores below the median.

The PNT profiles identified in this study enhance our understanding from previous research on psychological need satisfaction (PNS) and psychological need frustration (PNF) profiles (61–63). It’s important to note that within the context of Self-Determination Theory, PNT and PNF are distinct yet interrelated constructs (64). PNF typically relates to situations where one’s basic psychological needs remain unfulfilled (65). Continuous experiences of need frustration can result in feelings of ineffectiveness and a perceived loss of control, prompting individuals to adopt specific behaviors to regain autonomy and competence (66). Conversely, PNT is viewed as an active hindrance or disruption to one’s basic psychological needs. Therefore, this study augments, rather than replicates, existing PNF research.

### 4.2 Association of PNT with PSU, psychological distress, and perceived administrative support

In examining variable correlations, this study determines that, overall, the three PNTs are negatively correlated with perceived administrative support and positively with psychological distress and PSU at both time points. This is consistent with prior research (37, 67, 68). For example, Schultz et al. (67) found that reduced managerial support actively thwarts employees’ basic psychological needs.

Regarding PSU, SEM results reveal that at Time 1, Class 2’s score was higher than that of Class 1, followed by Class 3. This pattern indicates significant differences in PSU levels among teachers with varying degrees of PNT during the pandemic, with those having high PNT facing the highest risk. This finding is consistent with the Compensatory Internet Use Theory (CIUT), which suggests that people turn to smartphones to compensate for dysphoria and avoid real-life problems (35). It also aligns with prior studies (13, 14) showing that engaging in online social networks on smartphones helps individuals with PNT connect with others, providing a sense of relatedness and autonomy. Playing online games on smartphones fulfills the needs for social interaction (relatedness), achievement (competence), and decision-making (autonomy). Therefore, the higher the PNT level, the greater the likelihood of developing PSU. This explains the descending order of PSU severity among the three classes, ranked as 2, 1, and 3. However, at Time 2, no significant differences were observed between these groups, suggesting that the impact of PNT on PSU was most pronounced during the pandemic and lessened as the pandemic eased and negative emotions decreased.

Concerning psychological distress, the three profiles rank as follows at both time points: Class 2 > Class 1 > Class 3. This ranking reaffirms previous findings (1, 10, 12) that PNTs correlate positively with psychological distress. Past research has shown that thwarting of the three basic psychological needs can exacerbate negative outcomes such as burnout (1) and depression (10, 12). In terms of perceived administrative support, given that the profiles rank as C3 > C1 > C2 at both time points and considering the previously identified negative relationship between PNT and perceived administrative support, it can be concluded that relatedness thwarting plays a pivotal role in this domain, especially when considering the differences between Class 1 and 2. This study diverges from earlier findings (40), highlighting the significant impact of relatedness thwarting, as opposed to competence and autonomy thwarting. The analysis confirms a negative correlation between PNT and perceived administrative support, and a positive correlation with both psychological distress and PSU. Importantly, it identifies relatedness thwarting as a key factor negatively impacting perceived administrative support and positively affecting psychological distress, emphasizing its critical role in the framework of basic psychological needs.

### 4.3 Analysis of changes and influence of PNT through longitudinal study

The present longitudinal study shows a decrease in PSU and perceived support scores from Time 1 to Time 2, with the pandemic’s alleviation being a key predictor of this change. Echoing previous studies (6, 69), teachers faced high demands, overload, physical isolation, and scant administrative support during the pandemic, leading to increased reliance on PSU as a coping mechanism. With the pandemic’s easing and school reopening, there was a shift in focus toward teaching improvement and student attainment, reducing the need for compensatory smartphone use and lessening the demand for administrative support, hence the lower scores at Time 2. However, it’s important to recognize that reopening schools did not restore the pre-pandemic environment (70). New challenges, such as the necessity for ICT skills and resource imbalances, continued to contribute to teachers’ psychological distress. Interestingly, unlike previous reports of heightened psychological distress in teachers post-reopening, this study observed no significant difference in psychological distress levels at Time 2.

Regarding perceived administrative support, this study determined that based on the scores, the three groups were ranked as Class 3, Class 1, and then Class 2 at both time points. This suggests that both Class 3 and Class 1 perceived greater administrative support than Class 2. Likewise, in terms of psychological distress, the scores of the three groups are ordered as Class 2, Class 1 and Class 3 from high to low at both time points. It’s important to highlight that for Class 2, which exhibited high thwarting in autonomy, competence, and relatedness, perceived administrative support consistently remained low across both time points. In contrast, the psychological distress scores of this class occupies the highest position at Time 1 as well as Time 2. This suggests that high PNT has a sustained, longitudinal influence on these two variables. The observation presents a novel insight when contrasted with existing cross-sectional research on PNT (41, 42).

## 5 Limitations, future directions and conclusion

### 5.1 Limitations

The present study, while providing valuable insights, acknowledges key limitations including potential observer, recall, and time-related biases. The involvement of the city’s Education Bureau in our sampling process may limit the representativeness of our sample and introduce observer bias, as teachers’ responses could be influenced by the perceived authority of the data collectors. Additionally, our reliance on retrospective recall for the second measurement is susceptible to recall bias, potentially skewing the data due to teachers’ current circumstances influencing their recollections. A significant time-related bias arises from the study’s two distinct phases: mandatory online learning (Time 1) and offline teaching (Time 2). This shift in teaching modes could affect the comparability of data across these periods, introducing inconsistencies. Furthermore, the unique conditions of our study, particularly during the pandemic, may not accurately represent traditional educational settings, posing another limitation in terms of the generalizability of our findings. Moreover, although according to the aforementioned data provided by the Ministry of Education, there are more female teachers among primary and secondary school teachers, the overrepresentation of female teachers in this study is indeed a research limitation. Lastly, we did not explore some variables serving as mediators or moderators within our conceptual model (i.e., Figure 6) which is also a limitation of the present study.

### 5.2 Future directions

The limitations identified in our study pave the way for future research to validate our findings within more traditional educational settings, ensuring a balanced gender ratio to comprehensively evaluate their applicability and generalizability. Future sampling methods should aim to minimize the influence of authority to reduce potential data bias. Moreover, our study did not encompass the exploration of mediators or moderators. Therefore, future research could beneficially incorporate variables such as ‘anti-mattering’ into our testing model, as illustrated in Figure 6. Anti-mattering, the perception of being insignificant to others, often leads individuals to feel alienated and can increase susceptibility to mental health issues. Those exhibiting high levels of anti-mattering are more likely to rely on smartphones for interaction rather than engaging directly with others. Additionally, when individuals perceive themselves as irrelevant to others, they may overlook or fail to recognize the care and support available to them. Therefore, examining the mediating effects of anti-mattering could provide insightful contributions to our understanding of its impact in educational and social contexts.

### 5.3 Conclusion

To conclude, our present study is the first to apply a person-centered approach rather than a variable-centered model to investigate PNT. Three profiles are identified, and different associations are found between them and psychological distress, PSU, and perceived administrative support. A detailed understanding of teachers’ PNT status fosters the development of more targeted intervention measures, then to lessen teachers’ psychological distress, PSU, and increase perceived administrative support. Moreover, through longitudinal data, the present study demonstrates that PNT exerts a lasting influence, which complements findings from cross-sectional studies and highlights the importance of focusing on teachers’ basic psychological needs.

## Data availability statement

The raw data supporting the conclusions of this article will be made available by the authors, without undue reservation.

## Ethics statement

The studies involving humans were approved by Jiangxi Psychological Consultant Association (IRB ref.: JXSXL-2020-J013). The studies were conducted in accordance with the local legislation and institutional requirements. The participants provided their written informed consent to participate in this study.

## Author contributions

X-LL: Conceptualization, Investigation, Writing – original draft. C-HC: Conceptualization, Writing – original draft, Writing – review & editing. JG: Writing – review & editing. L-LL: Data curation, Writing – review & editing. X-YJ: Data curation, Writing – review & editing. C-XB: Data curation, Funding acquisition, Writing – review & editing. I-HC: Formal analysis, Methodology, Software, Supervision, Writing – original draft, Writing – review & editing.
