# Supplementary material for: Latent profile analysis of psychological needs thwarting in Chinese school teachers: longitudinal associations with problematic smartphone use, psychological distress, and perceived administrative support
Source: Front Public Health. 2023 Dec 18;11:1299929. doi: 10.3389/fpubh.2023.1299929 (PMC10764027; doi:10.3389/fpubh.2023.1299929)
Supplement: Supplementary file 1 [file Data_Sheet_1.docx]

Table S1: Four measures of both English and Chinese versions

1. Psychological Need Thwarting Scale of Online Teaching (PNTSOT) 线上教学心理需求挫败量表

| **Subscale of autonomy** | **自主挫败** |
| --- | --- |
| 1. In online courses during the pandemic, I cannot decide for myself how I want to teach. | 1. 此期间的线上课程进行时，我无法自己决定想要的教学方式。 |
| 2. In online teaching work during the pandemic, I feel there is pressure that affects my behavior and requires me to comply in a certain way. | 2. 线上教学工作进行时，我觉得有股压力会影响我的行为举止，使其符合特定规范。 |
| 3. I have to follow a prescribed online teaching style during the pandemic. | 3. 我必需遵循某种规定的线上教学方式。 |
| 4. During the pandemic, I feel pressure from the external environment that limited me in choosing a particular online teaching style. | 4. 我感受到外在环境的压力，限制我必须选定特定的线上教学方式。 |
| **Subscale of competence** | **能力挫败** |
| 5. There are some online teaching situations that make me feel incapable in my daily work environment during the pandemic. | 5. 有些线上教学的情况让我觉得无能为力。 |
| 6. I sometimes talk about the things that make me feel powerless to do my online teaching job during the pandemic. | 6. 我有时会跟人提到线上教学工作中让我感到无能为力的事情。 |
| 7. Online teaching during the pandemic sometimes makes me feel powerless. | 7. 线上教学工作有时会让我产生无力感。 |
| 8. Due to the lack of training opportunities in my environment, I feel that I am capable of performing online teaching tasks. | 8. 由于环境中缺乏磨练机会，我觉得自己不能胜任线上教学的工作任务。 |
| **Subscale of relatedness** | **关系挫败** |
| 9. I feel disconnected from other colleagues and leaders when teaching online during the pandemic. | 9. 进行线上教学时，我觉得自己与其他同事及领导之间有所隔阂。 |
| 10. I do not feel that my colleagues and leaders care about me when teaching online during the pandemic. | 10. 线上教学进行时，我无法感受到同事及领导对我的关心。 |
| 11. I feel that my colleagues and leaders are jealous of me when I achieve good results in online teaching during the pandemic. | 11. 线上教学取得良好成效时，我觉得同事与领导会嫉妒我。 |
| 12. I feel that my colleagues and leaders do not like me when I conduct online teaching during the pandemic. | 12. 进行线上教学时，我觉得同事与领导不喜欢我。 |

2. Smartphone Application-Based Addiction Scale (SABAS) 智能手机成瘾量表

| 1. My smartphone is the most important thing in my life. | 1. 我的智能手机是我生活中最重要的东西。 |
| --- | --- |
| 2. Conflicts have arisen between me and my family (or friends) because of my smartphone use. | 2. 我曾因为智能手机的使用，而和我家人或朋友发生冲突。 |
| 3. Preoccupying myself with my smartphone is a way of changing my mood (I get a buzz, or I can escape or get away, if I need to). | 3. 专注使用智能手机是改变我心情的一个途径（从中感到开心或在有需要的时候借此逃避）。 |
| 4. Over time, I fiddle around more and more with my smartphone. | 4. 从开始玩手机起，我玩智能手机的时间越来越长。 |
| 5. If I cannot use or access my smartphone when I feel like, I feel sad, moody, or irritable. | 5. 当我想玩智能手机却拿不到或玩不成时，我会感到想发脾气，生气、难受或心烦。 |
| 6. If I try to cut the time I use my smartphone, I manage to do so for a while, but then I end up using it as much or more than before. | 6. 若我尝试减少使用智能手机的时间，我只能坚持很短一段时间，最终只会跟以前一样，甚至比当初使用得更多。 |

3. The Depression, Anxiety, and Stress Scale-21 Version 抑郁、焦虑和压力量表（21条版本）

| 1. I found it hard to wind down. | 1. 我觉得很难让自己安静下来。 |
| --- | --- |
| 2. I was aware of dryness of my mouth. | 2. 我感到口干。 |
| 3. I couldn’t seem to experience any positive feeling at all. | 3. 我好像不能再有任何愉快、舒畅的感觉。 |
| 4. I experienced breathing difficulty (e.g., excessively rapid breathing, breathlessness in the absence of physical exertion). | 4. 我感到呼吸困难（例如不是做运动时也感到气促或透不过气来）。 |
| 5. I found it difficult to work up the initiative to do things. | 5. 我感到很难自动去开始工作。 |
| 6. I tended to over-react to situations | 6. 我对事情往往作出过敏反应。 |
| 7. I experienced trembling (e.g., in the hands). | 7. 我感到颤抖（例如手震）。 |
| 8. I felt that I was using a lot of nervous energy | 8. 我觉得自己消耗很多精神。 |
| 9. I was worried about situations in which I might panic and make a fool of myself. | 9. 我忧虑一些令自己恐慌或出丑的场合。 |
| 10. I felt that I had nothing to look forward to. | 10. 我觉得自己对将来没有什么可盼望。 |
| 11. I found myself getting agitated. | 11. 我感到忐忑不安。 |
| 12. I found it difficult to relax. | 12. 我感到很难放松自己。 |
| 13. I felt down-hearted and blue. | 13. 我感到忧郁沮丧。 |
| 14. I was intolerant of anything that kept me from getting on with what I was doing. | 14. 我无法容忍任何阻碍我继续工作的事情。 |
| 15. I felt I was close to panic. | 15. 我感到快要恐慌了。 |
| 16. I was unable to become enthusiastic about anything. | 16. 我对任何事也不能热衷。 |
| 17. I felt I wasn't worth much as a person. | 17. 我觉得自己不怎么配做人。 |
| 18. I felt that I was rather touchy. | 18. 我发觉自己很容易被触怒。 |
| 19. I was aware of the action of my heart in the absence of physical exertion (e.g., sense of heart rate increase, heart missing a beat) | 19. 我察觉自己在没有明显的体力劳动时，也感到心律不正常。 |
| 20. I felt scared without any good reason. | 20. 我无缘无故地感到害怕。 |
| 21. I felt that life was meaningless. | 21. 我感到生命毫无意义。 |

4. Teachers’ Perception of Administrators’ Support Scale 教师对管理者支持的认知量表

| 1. Administrators want teachers to be able to teach online smoothly during the outbreak. | 1. 领导者希望教师能顺利地在疫情期间进行线上教学。 |
| --- | --- |
| 2. School administrators provided most of the necessary resources to help teachers be able to teach online during the outbreak. | 2. 学校管理部门提供了大部分必要的资源，帮助教师能在疫情期间进行线上教学。 |
| 3. Administrators always support and encourage teachers to use online teaching during the pandemic. | 3. 领导者始终支持和鼓励教师于疫情期间使用线上教学。 |
| 4. Administrators understand the benefits of using online teaching during the pandemic. | 4. 领导者了解疫情期间教师使用线上教学所带来的好处。 |
